# Dynamic effects of fermentation on phytochemical composition and antioxidant properties of wampee (*Clausena lansium* (Lour.) Skeel) leaves

**DOI:** 10.1002/fsn3.795

**Published:** 2018-11-19

**Authors:** Quan Li, Xiaoxiao Chang, Ruixue Guo, Qijun Wang, Xinbo Guo

**Affiliations:** ^1^ School of Food Science and Engineering South China University of Technology Guangzhou China; ^2^ Institute of Fruit Tree Research Guangdong Academy of Agricultural Sciences Guangzhou China

**Keywords:** antioxidant activity, fermentation, phytochemicals, wampee leaves

## Abstract

Variations in the phytochemical composition and antioxidant properties were studied in the wine of wampee leaves obtained at different stages of fermentation process. The highest concentrations of total phenolic and flavonoid contents were attained at Day 12 and Day 21 of fermentation, respectively. In addition, five phytochemical compounds including vanillic acid, *p*‐coumaric acid, rutin, ferulic acid, and 7‐hydroxycoumarin were identified and quantified by HPLC in fermented wampee products. The strongest antioxidant activity in wine was monitored on Day 12. Furthermore, total antioxidant activity was significantly correlated with vanillic acid, *p*‐coumaric acid, ferulic acid, and 7‐hydroxycoumarin compared with rutin. The obtained results suggested that 12‐day fermentation could be an optimal process for excavation of applying wampee leaves into food and wine industries.

## 1 INTRODUCTION

Microbial strategies have attracted an increasing attention in wine‐making from various fruits, that is grape, pomegranate, apple, mango, and grains including polished glutinous rice and maize (Chang, Jang, Lin, & Duan, 2016; Lim, Jeong, & Shin, 2012; Lu, Chan, Li, & Liu, 2018). The levels of volatile compounds and their health benefits have been enhanced in the wine of Korean black raspberry *Rubus coreanus* Miq. using modified fermentation process (Lim et al., 2012). Likewise, significant levels of free radical scavenging activity and antimicrobial potential have been reported in the wine of pomegranate and commercial rice (Chang et al., 2016). The fermentation process combined with UV‐B treatment had considerably enhanced the nutritional value in the fermented leaves of white cabbage (Harbaum‐Piayda, Palani, & Schwarz, 2016), polyphenolics in black tea (Kerio, Wachira, Wanyoko, & Rotich, 2013), and antioxidant capacity of yellow tea (Gramza‐Michałowska et al., 2016). In addition, Lu reported highest levels of volatile compounds notably ethyl esters, acetate esters, and isoamyl alcohol in mango wine (Lu et al., 2018).

Wampee (*Clausena lansium* (Lour.) Skeel) belongs to the Rutaceae family are being extensively cultivated in South of China. It attracts multitudinous consumers because of special taste and folk medical applications (Prasad et al., 2010). In the current decade, wampee leaves have gained substantial attention by consumers and researchers (Sciarrone et al., 2013). Traditionally, wampee leaves have been reported to treat asthma, cough, and dermatological problems (Liu et al., 2014). Additionally, it has been reported that wampee leaves had antiacetylcholinesterase (anti‐AChE) activity that could be applied to cure Alzheimer's disease (Huang, Cai, Corke, & Sun, 2010; Shen et al., 2015; Tang & Zhang, 2003). Previous investigations have revealed that the antioxidant and antimicrobial activities of wampee leaves are mainly attributed to high contents of flavonoids and phenolics (Du et al., 2015; Prasad et al., 2010).

With the aim of further expanding the application of wampee leaves in food industries, we prepared wampee leaves wine (WLW) for the first time in the present study. Furthermore, dynamic changes in the concentration of phenolics, flavonoids, and antioxidant activities were also studied in WLW along with alcoholic composition during the process of fermentation at different intervals.

## 2 MATERIALS AND METHODS

### 2.1 Chemicals and reagents

Folin–Ciocalteu reagent, dichlorofluorescin diacetate, 2,2′‐Azobis (2‐methylpropionamidine) dihydrochloride, vanillic acid, *p*‐coumaric acid, rutin, ferulic acid, and 7‐hydroxycoumarin were purchased from Sigma‐Aldrich (MO, USA). Yeast (*Saccharomyces cerevisiae*) was supplied by Angel Yeasts Co., Ltd. (Hubei, China). Cellulase and pectinase were purchased from Shanghai Yuanye Biotechnology Co., Ltd. (Shanghai, China). Other materials used in wine‐making were of food grade. All chemicals and regents used in this work were of analytical grade.

### 2.2 Fermentation of wampee leaves

**Figure 1:**
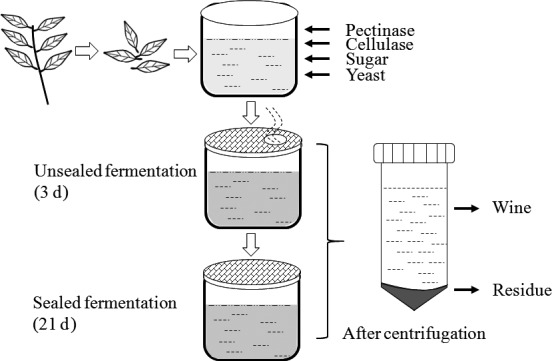
Preparation of wampee leaves wine (WLW). The contents of pectinase, cellulase, sugar, and yeast in leaves juice were 0.005%, 0.01%, 21%, and 0.02%. Samples were collected every 3 days since unsealed fermentation

Wampee leaves were obtained from the institute of Fruit Tree Research, Guangdong Academy of Agricultural Sciences. Fermentation process was conducted according to a method described previously (Tang, Zeng, Brennan, & Xie, 2017) with slight modification in our laboratory (Figure 1). Briefly, fresh wampee leaves were cleaned with distilled water, then crushed using distilled water in a mass ratio of 1:9. Cellulase, pectinase, and sugar were added in leaves juice up to the concentrations of 0.01%, 0.005%, and 21%, respectively. Yeast (0.02%) was inoculated in well‐mixed juice, and the juice was transferred to an unsealed glass vessel then stored at 25°C for 3 days. After 3 days of unsealed fermentation, the juice was fermented at 20°C in a sealed vessel for 21 days. The first sample was collected at the time of yeast inoculation (Day 0) and other samples were collected after every 3rd day of fermentation after thorough mixing fermented fluid. All samples were centrifuged immediately after collection and supernatant and precipitant were graded as WLW and residue, respectively. All samples were stored at −20°C until analyzed. Distilled water dealt with the same procedure was used as blank control.

### 2.3 Headspace gas chromatography (HS‐GC)

Headspace gas chromatographic (HS‐GC) technique as explained earlier (Tang et al., 2017) was operated to analyze volatile acids and alcoholic compounds in WLW. In short, 5 ml of WLW was placed in a headspace container of 21.6 ml, sealed and evaluated by a GC system (Agilent 7820A, USA) equipped with FID, HP‐5 capillary column (30 m × 0.25 mm × 0.25 mm, Agilent, USA) and automatic headspace sampler (HSS 86.50 PLUS, DANI, Italy). And nitrogen was used as the makeup gas. The headspace procedure for WLW was as follows: 30 min of shaking was carried out at 50°C, needle and sampling coil were preheated to 70°C, transfer line was preheated to 80°C, 0.5 ml was used as the default sample size, 2.00 bars of pressurization pressure, 1.5 bar of carrier gas pressure, 15 s of vial pressurization time, 10 s of sample loop fill time, and 20 s of transfer time. The injection port was kept at 230°C, FID was at 250°C; flow of nitrogen was at 30 ml/min. Program of temperature was accomplished at 40°C. Then, the temperature was held for 4 min at 40°C, raised to 210°C at 10°C/min, and maintained at 210°C for 10 min. The split ratio was 20:1. Results were presented as gram per liter (g/l) in triplicates.

### 2.4 Extraction of polyphenolics

To extract polyphenolic compounds, the wampee leaves wine was concentrated and redissolved in distilled water. The free and bound polyphenolics in WLW and residue were extracted according to the method as reported (Wang et al., 2016). All samples were stored at −20°C until analysis.

### 2.5 Determination of phenolic and flavonoid contents

The free bound and total phenolic content (TPC) in WLW and residue were estimated following the procedure as reported before (Wang et al., 2016). Results were presented as milligram gallic acid equivalents per 100 g (mg GAE/100 g) for WLW and residue. Likewise, free, bound, and total flavonoid contents (TFC) were also determined as explained by Wang et al. (2016) and results were presented as milligram catechin equivalents per 100 g (mg CE/100 g) for WLW and residue.

### 2.6 Quantification of phenolic acids and flavonoids by HPLC

Phenolic acids and flavonoids in WLW and residue were executed referring to the former study (Guo, Guo, Li, Fu, & Liu, 2017) with slight modification. Briefly, samples were evaluated by HPLC technique with Waters 2998 Photodiode Array Detector at 280 and 370 nm wavelengths with a C18 column (250 × 4.6 mm, 5 μm) maintained at 35°C. The flow rate of the mobile phases (A: 0.1% trifluoroacetic acid in water, B: acetonitrile) was 1.0 ml/min in gradient elution of: 0–5 min (90% A), 5–20 min (90–75% A), 20–25 min (75–65% A), 25–31 min (65–42% A), 31–34 min (42–40% A), 34–40 min (40–10% A), 40–50 min (10–90% A), and 50–60 min (90–90% A). Measured values were presented as milligram per 100 g of samples (mg/100 g) for WLW and residue.

### 2.7 Antioxidant activity assays

Antioxidant property of WLW and residue was examined using peroxyl radical scavenging capacity (PSC) assay as described previously (Wang, Sun, et al., 2015; Wang, Wang, et al., 2015). Results were expressed as micromoles of ascorbic acid (ASA) equivalent per 100 g (μmol ASA equiv./100 g) for WLW and residue.

### 2.8 Statistical analysis

Data were presented as mean ± standard deviation (*n* = 3). Measured levels of phytochemical content and antioxidant capacity were analyzed by one‐way analysis of variance (ANOVA) and Ducan's multiple comparison post‐test in SPSS statistical software 21.0 (SPSS Inc., Chicago, IL, USA) at a significant level with *p*‐value < 0.05.

## 3 RESULTS AND DISCUSSION

### 3.1 Change in the alcoholic composition of WLW

Ethanol, iso‐butanol, acetic acid, and butyric acid were quantified by HS‐GC in WLW (Table 1). Methanol, propanol, and active amyl alcohol were below the detection limit, while iso‐butanol was only detected in the start of sealed fermentation. Butyric acid appeared in Day 3 to 9 of fermentation process with a decreasing tendency. Besides, WLW presented a significantly higher ethanol content compared with control. Ethanol content of WLW showed an increasing trend during the fermentation and was highest at 94.32 ± 2.71 g/L on Day 24 compared to initial concentration 0.29 ± 0.02 g/L. Moreover, the content of acetic acid changed from 1.62 ± 0.11 to 5.55 ± 0.37 g/L during first 3 days then kept down to 0.76 ± 0.06 g/L on Day 24. The acetic acid content of WLW was recorded after Day 18 fermentation compared with control.

**Table 1:** Dynamic changes in ethanol, butanol, acetic acid, and butyric acid (g/L)during alcoholic fermentation

	Unsealed fermentation		Sealed fermentation						
	Day 0	Day 3	Day 6	Day 9	Day 12	Day 15	Day 18	Day 21	Day 24
Ethanol									
Control	ND	ND	0.89 ± 0.03^g^	1.88 ± 0.04^f^	2.64 ± 0.08^e^	3.52 ± 0.06^d^	3.79 ± 0.13^c^	5.00 ± 0.12^b^	4.80 ± 0.19^a^
WLW	0.29 ± 0.02^g^	35.05 ± 1.58^f^	36.63 ± 2.17^f^	46.33 ± 2.34^e^	57.76 ± 1.11^d^	56.70 ± 1.42^d^	71.33 ± 1.42^c^	89.69 ± 3.09^b^	94.32 ± 2.71^a^
Iso‐butanol									
Control	ND	ND	ND	ND	ND	ND	ND	ND	ND
WLW	ND	ND	0.06 ± 0.01	ND	ND	ND	ND	ND	ND
Acetic acid									
Control	ND	ND	ND	2.38 ± 0.08^d^	2.57 ± 0.07^c^	2.64 ± 0.12^c^	2.79 ± 0.06^b^	2.94 ± 0.03^a^	2.88 ± 0.07^ab^
WLW	1.62 ± 0.11^e^	5.55 ± 0.37^a^	3.58 ± 0.17^b^	3.29 ± 0.13^bc^	3.33 ± 0.15^bc^	3.06 ± 0.22^c^	1.98 ± 0.06^d^	0.96 ± 0.01^f^	0.76 ± 0.06^f^
Butyric acid									
Control	ND	ND	ND	ND	ND	ND	ND	ND	ND
WLW	ND	0.35 ± 0.01^a^	0.28 ± 0.03^b^	0.11 ± 0.02^c^	ND	ND	ND	ND	ND

*Notes*. Data are presented as means ± standard deviation (*n* = 3). “WLW” means wampee leaves wine. Different letters (a‐g) in the same row indicate significant differences at *p* < 0.05 from different wine samples. Methanol, propanol, and active amyl alcohol did not be detected in this study.

Preproduction of ethanol level depends on type of raw materials. Such as 184.0 and 150.0 g/L enhancement in the ethanol content was reported in Chinese rice wine and red wine (Frost, Harbertson, & Heymann, 2017; Yang, Xia, Wang, Yu, & Ai, 2017). The presented fluctuation of ethanol in WLW was consistent with ponkan wine (Lee, Chang, Yu, Lai, & Lin, 2013). The level of ethanol in WLW was up to 94.32 ± 2.71 g/L on Day 24 with the initial sugar concentration of 21%, while the ethanol content on Day 24 in ponkan wine was higher than 100.0 g/L with the initial sugar concentration of 24%, which could be related to proportional alcohol generation as a result of sugar consumption (Lee et al., 2013). Methanol is notorious in wine products because of depressant effects on central nervous system (Taheri, Moghaddam, Moharamzad, Dadgari, & Nahvi, 2010). Methanol is generated from enzymatic degradation of natural pectic substances, which have often been observed in wines and distilled beverages (Cabaroglu, 2005). However, in the present study, methanol was not detected during wine‐making process according to GC data. Matured leaves and the concentration of leaves–liquid ratio (1:10) in wine‐making procedure probably be the critical points of methanol production, because modifications of pectin in matured leaves were more limited than other sources such as stem and fruits (Bédouet, Denys, Courtois, & Courtois, 2006) or just maybe related to undetectable low level. Acetic acid could provide a “floral‐sweaty” odor to wine with other volatile compounds, which is usually linked to wine spoilage produced by acetic acid bacteria with abundant oxygen (Roda et al., 2017). The level of acetic acid in WLW was highest on Day 3 (5.55 ± 0.37 g/L) compared to the initial concentration of 1.62 ± 0.11 g/L, furthermore decreased up to 0.76 ± 0.06 g/L on Day 24. The unsealed fermentation did not promote the growth of yeasts on the first 3 days, however, improved the production of acetic acid. Considering the extraordinary stability of acetic acid (Li et al., 2018), the decline should not be imputed to natural degradation. Acetic acid could be a carbon source for yeasts in lipid‐production (Huang, Shen, Luo, Liu, & Liu, 2018), same may probably be happened in WLW fermentation. Fusel alcohols such as propanol and active amyl alcohol contribute positively to wine aroma properties (de‐la‐Fuente‐Blanco, Sáenz‐Navajas, & Ferreira, 2016). However, the levels of these two compounds were undetectable in WLW. The glimpse of iso‐butanol in WLW on Day 6 (up to detectable level in the beginning of sealed fermentation while decreased rapidly to undetectable level) might be due to the degradation of iso‐butanol, which was consistent with the decrease in iso‐butanol as reported in bog bilberry syrup wines (Wang et al., 2018). Meanwhile, butyric acid was generated in unsealed fermentation and degraded to undetectable concentration after 9 days of sealed fermentation, which probably caused by microbial activity.

### 3.2 Dynamic changes in phenolic and flavonoid contents

Measured levels of free, bound, and total phenolic contents (TPC) in WLW and residue were significantly different (*p *<* *0.05) in all nine samples as shown in Table 2. The TPC level in WLW was highest on Day 12 (105.3 ± 1.9 mg GAE/100 g), whereas lowest value TPC was observed on Day 0 (90.52 ± 3.11 mg GAE/100 g). This shows that fermentation process could promote phenolic content in the case of residue, the highest free phenolics at 137.4 ± 4.6 mg GAE/100 g and bound phenolics at 27.65 ± 2.24 mg GAE/100 g were noted on Day 0 and Day 21, respectively. And the lowest free (108.2 ± 6.1 mg GAE/100 g) and bound (20.84 ± 1.69 mg GAE/100 g) phenolics content was recorded on Day 6 and Day 0, respectively. In addition, TPC in residue was maximum on Day 0 (158.3 ± 5.2 mg GAE/100 g), while minimum on Day 12 (134.4 ± 10.5 mg GAE/100 g). Furthermore, enhancement in the phenolic content was observed in WLW obtained through fermentation compared to initial juice. So, 12‐day fermentation would be the best time for maximum yield of TPC in WLW.

**Table 2:** Change in the phenolic (mg GAE/100 g) and flavonoid contents (mg CE/100 g) of WLW and residue

Name	Sample	Unsealed fermentation		Sealed fermentation						
		Day 0	Day 3	Day 6	Day 9	Day 12	Day 15	Day 18	Day 21	Day 24
Phenolics	WLW	90.52 ± 3.11^e^	96.64 ± 0.89^bcd^	99.75 ± 1.12^bc^	95.77 ± 2.82^cd^	105.3 ± 1.9^a^	101.2 ± 4.7^ab^	96.35 ± 1.24^bcd^	92.34 ± 2.48^de^	94.39 ± 2.95^de^
	FR	137.4 ± 4.6^h^	119.6 ± 2.8^ij^	108.2 ± 6.1^j^	109.2 ± 5.1^ij^	108.9 ± 10.4^j^	109.4 ± 5.6^ij^	121.2 ± 9.5^i^	121.3 ± 6.1^i^	117.8 ± 2.1^ij^
	BR	20.84 ± 1.69^p^	25.51 ± 2.07^o^	27.19 ± 2.06^o^	27.55 ± 1.55^o^	25.55 ± 0.41^o^	27.53 ± 0.75^o^	26.33 ± 1.19^o^	27.65 ± 2.24^o^	25.94 ± 1.72^o^
	TR	158.3 ± 5.2^u^	145.2 ± 0.8^vw^	135.4 ± 8.0^w^	136.7 ± 6.4^vw^	134.4 ± 10.5^w^	137.0 ± 6.0^vw^	147.5 ± 8.7^uvw^	149.0 ± 8.2^uv^	143.8 ± 2.6^vw^
Flavonoids	WLW	26.06 ± 2.18^d^	29.80 ± 1.00^c^	32.46 ± 2.38^abc^	31.2 ± 2.24^bc^	31.55 ± 2.41^bc^	32.27 ± 1.87^abc^	34.46 ± 2.05^ab^	35.94 ± 2.76^a^	34.36 ± 2.19^ab^
	FR	75.42 ± 6.55^h^	62.24 ± 3.15^i^	49.61 ± 4.00^j^	47.68 ± 3.63^j^	44.7 ± 3.18^j^	38.66 ± 3.43^k^	33.02 ± 1.85^kl^	30.42 ± 1.83^d^	28.12 ± 0.34^d^
	BR	85.67 ± 4.29^o^	83.04 ± 3.24^o^	82.6 ± 7.22^o^	67.16 ± 5.86^p^	64.08 ± 4.38^p^	61.81 ± 2.78^p^	50.10 ± 3.55^q^	48.53 ± 1.38^q^	50.35 ± 3.72^q^
	TR	161.1 ± 4.0^u^	145.2 ± 6.0^v^	132.2 ± 8.3^w^	114.8 ± 9.5^x^	108.8 ± 7.5^xy^	100.5 ± 5.8^y^	83.12 ± 5.40^z^	78.95 ± 3.16^z^	78.46 ± 4.03^z^

*Notes*. Data are presented as means ± standard deviation (*n* = 3). “WLW” means wampee leaves wine, “FR” means free residue, “BR” means bound residue, and “TR” means total residue. Different letters in the same row indicate significant differences at *p* < 0.05 from different wine samples. Letters group “a–g” corresponds to the row of wine, “h–n” corresponds to the row of free in residue, “o–t” corresponds to the row of bound in residue, and “u–z” corresponds to the row of total form in residue. Phenolic content and flavonoid content of control did not be detected.

Being an important member of antioxidants presented in plants, phenolic compounds are attracting an increasing attention for prevention and management of chronic diseases caused by free radicals (Guo et al., 2017). Recently, Chen reported free, esterified, insoluble‐bound, and total phenolic contents of wampee leaves, which were 4.49, 3.09, 7.07, and 14.65 mg GAE/g DW, respectively (Chen, Zhang, Chen, Han, & Gao, 2017). Although insoluble‐bound phenolic content was dominant fraction, the presence of strong covalent bound between insoluble‐bound phenolic and cell wall restricted to the direct application of wampee leaves extract in folk medicine such as to treat cough. In the present work, slight increase was noted in the TPC of WLW from Day 0 to Day 12. This increase was roughly stable with a mildly fluctuation. Similar alternation was observed during mulberry wine‐making (Wang, Sun, et al., 2015; Wang, Wang, et al., 2015), where enhancement in TPC was observed during the first three days of mulberry wine fermentation. Afterward, a stability or mild decrease was noted from Day 3 to Day 10. This could be explained by the fact that phenolic extraction could be influenced by the variation of hydro‐alcoholic medium during alcoholic fermentation (Di Egidio, Sinelli, Giovanelli, Moles, & Casiraghi, 2010). Phenolics extracted from wampee leaves exhibited an increasing trend which could be due to the increasing alcohol content in the start of fermentation (Day 0 to Day 3) in WLW. Moreover, stability or mildly fluctuation in TPC was probably due to the oxidation and hydrolysis of the original phenolics (Chen et al., 2017).

Dynamic changes in the total flavonoids content (TFC) of WLW and residue were presented in Table 2. In wampee leaves, wine highest concentration of TFC was calculated on Day 21, while lowest on Day 0 at 35.94 ± 2.76 and 26.06 ± 2.18 mg CE/100 g, respectively. These values were significantly different at *p *<* *0.05. In residue, TFC ranged between 78.46 ± 4.03 and 161.1 ± 4.0 mg CE/100 g on Day 0 and Day 24, respectively. The maximum and minimum bound flavonoid contents were calculated on Day 0 and Day 21, respectively.

In WLW, change in TFC was similar to TPC and was consistent with previous reports in mulberry wine (Wang, Sun, et al., 2015; Wang, Wang, et al., 2015). This revealed that the yield of flavonoids from wampee leaves was mainly attributed to higher alcohol content. Likewise, considerable changes in free and bound flavonoid contents were also observed in residue. This may influence by increasing hydro‐alcoholic medium, oxidation process, and microbe activity. Although TFC of WLW enhanced during fermentation, bound flavonoids in residue were dominant which indicated that further studies should focus on higher fractional conversions of bound form into easy‐accessible forms.

### 3.3 Changes in the phytochemical composition of WLW and residue

Results of vanillic acid, *p*‐coumaric acid, rutin, ferulic acid, and 7‐hydroxycoumarin concentration determined in WLW and residue are given in Table 3. The descending order of these phytochemicals in WLW was: rutin > vanillic acid > 7‐hydroxycoumarin > ferulic acid > *p*‐coumaric acid. Rutin was the main flavonoid in WLW and was almost stable during fermentation process. An enhancement in the concentration of vanillic acid was observed in WLW from 3.66 ± 0.29 to 4.68 ± 0.14 mg/100 g on Day 6; however, it decreased to 4.13 ± 0.08 mg/100 g on Day 24.

**Table 3:** Variations in the phytochemical composition (mg/100 g)of WLW and residue

Stage	Date	Phytochemicals	WLW	FR	BR	TR
Unsealed fermentation	Day 0	Vanillic acid	3.66 ± 0.29^d^	4.93 ± 0.18^j^	1.52 ± 0.11^qrs^	6.45 ± 0.28^w^
		*p*‐Coumaric acid	0.43 ± 0.04^g^	0.75 ± 0.03^d^	3.97 ± 0.28^o^	4.72 ± 0.25^y^
		7‐Hydroxycoumarin	2.10 ± 0.16^b^	4.25 ± 0.34^h^	0.72 ± 0.05^r^	4.96 ± 0.32^v^
		Ferulic acid	1.11 ± 0.05^e^	2.03 ± 0.12^d^	3.99 ± 0.06^o^	6.02 ± 0.09^vw^
		Rutin	21.64 ± 1.29^a^	37.67 ± 2.15^h^	0.98 ± 0.08^op^	38.65 ± 2.18^u^
	Day 3	Vanillic acid	4.21 ± 0.03^bc^	5.51 ± 0.24^i^	1.54 ± 0.05^pqr^	7.05 ± 0.27^vw^
		*p*‐Coumaric acid	0.92 ± 0.08^f^	1.38 ± 0.09^k^	3.48 ± 0.14^p^	4.87 ± 0.21^xy^
		7‐Hydroxycoumarin	2.46 ± 0.18^a^	3.77 ± 0.09^i^	1.18 ± 0.09^p^	4.95 ± 0.06^v^
		Ferulic acid	1.45 ± 0.06^d^	2.30 ± 0.06^jk^	3.57 ± 0.12^pq^	5.87 ± 0.18^w^
		Rutin	20.81 ± 2.03^a^	29.17 ± 0.20^1j^	0.99 ± 0.05^o^	30.16 ± 0.23^w^
Sealed fermentation	Day 6	Vanillic acid	4.68 ± 0.14^a^	5.37 ± 0.35^ij^	1.62 ± 0.09^opq^	7.00 ± 0.32^vw^
		*p*‐Coumaric acid	1.03 ± 0.07^ef^	1.67 ± 0.13^j^	3.54 ± 0.29^op^	5.21 ± 0.27^wx^
		7‐Hydroxycoumarin	2.53 ± 0.01^a^	3.78 ± 0.35^i^	1.15 ± 0.09^p^	4.93 ± 0.40^v^
		Ferulic acid	1.59 ± 0.02^cd^	2.23 ± 0.04^kl^	3.30 ± 0.13^q^	5.53 ± 0.14^x^
		Rutin	22.16 ± 0.38^a^	27.92 ± 0.62^j^	0.97 ± 0.03^opq^	28.89 ± 0.60^w^
	Day 9	Vanillic acid	4.35 ± 0.14^bc^	5.25 ± 0.12^ij^	1.71 ± 0.05^op^	6.95 ± 0.13^vw^
		*p*‐Coumaric acid	1.02 ± 0.09^ef^	1.61 ± 0.11^j^	3.91 ± 0.31^o^	5.52 ± 0.25^vw^
		7‐Hydroxycoumarin	2.43 ± 0.09^a^	3.58 ± 0.24^ij^	1.30 ± 0.10^p^	4.88 ± 0.30^v^
		Ferulic acid	1.60 ± 0.03^cd^	2.18 ± 0.10^kl^	3.96 ± 0.15^o^	6.14 ± 0.07^vw^
		Rutin	21.18 ± 0.27^a^	28.07 ± 1.02^j^	0.89 ± 0.05^r^	28.96 ± 1.03^w^
	Day 12	Vanillic acid	4.40 ± 0.09^b^	5.56 ± 0.30^i^	1.75 ± 0.09^o^	7.32 ± 0.35^uv^
		*p*‐Coumaric acid	1.14 ± 0.10^de^	2.00 ± 0.09^i^	3.94 ± 0.21^o^	5.93 ± 0.19^u^
		7‐Hydroxycoumarin	2.55 ± 0.10^a^	4.29 ± 0.32^h^	1.56 ± 0.04^o^	5.85 ± 0.33^u^
		Ferulic acid	1.71 ± 0.12^bc^	2.87 ± 0.15^h^	3.85 ± 0.33^op^	6.72 ± 0.27^u^
		Rutin	22.01 ± 0.73^a^	29.44 ± 2.18^j^	0.88 ± 0.02^qr^	30.32 ± 2.16^w^
	Day 15	Vanillic acid	4.43 ± 0.05^b^	6.17 ± 0.49^h^	1.70 ± 0.12^opq^	7.87 ± 0.58^u^
		*p*‐Coumaric acid	1.19 ± 0.04^cd^	2.10 ± 0.12^i^	3.56 ± 0.12^op^	5.66 ± 0.22^uv^
		7‐Hydroxycoumarin	2.48 ± 0.19^a^	4.56 ± 0.13^h^	1.30 ± 0.11^p^	5.86 ± 0.24^u^
		Ferulic acid	1.73 ± 0.11^bc^	2.97 ± 0.19^h^	3.98 ± 0.23^o^	6.95 ± 0.06^u^
		Rutin	21.83 ± 0.89^a^	33.38 ± 0.88^i^	0.90 ± 0.04^pqr^	34.28 ± 0.86^v^
	Day 18	Vanillic acid	4.31 ± 0.09^bc^	5.68 ± 0.12^i^	1.36 ± 0.10^s^	7.04 ± 0.21^vw^
		*p*‐Coumaric acid	1.30 ± 0.08^c^	2.09 ± 0.09^i^	3.23 ± 0.23^p^	5.32 ± 0.14^vw^
		7‐Hydroxycoumarin	2.42 ± 0.22^a^	3.35 ± 0.03^ijk^	1.16 ± 0.06^p^	4.51 ± 0.06^vw^
		Ferulic acid	1.88 ± 0.16^ab^	2.61 ± 0.08^i^	3.73 ± 0.11^op^	6.33 ± 0.15^v^
		Rutin	20.72 ± 0.54^a^	23.57 ± 1.11^k^	0.76 ± 0.04^s^	24.33 ± 1.10^x^
	Day 21	Vanillic acid	4.26 ± 0.12^bc^	5.39 ± 0.30^ij^	1.38 ± 0.11^rs^	6.77 ± 0.40^vw^
		*p*‐Coumaric acid	1.43 ± 0.09^b^	2.40 ± 0.11^h^	3.16 ± 0.21^p^	5.56 ± 0.24^uvw^
		7‐Hydroxycoumarin	2.39 ± 0.12^a^	3.25 ± 0.16^jk^	0.91 ± 0.08^q^	4.16 ± 0.14^wx^
		Ferulic acid	1.85 ± 0.03^ab^	2.55 ± 0.10^i^	3.68 ± 0.28^op^	6.23 ± 0.19^v^
		Rutin	20.93 ± 1.36^a^	20.17 ± 1.14^l^	0.78 ± 0.05^s^	20.95 ± 1.10^y^
	Day 24	Vanillic acid	4.13 ± 0.08^c^	5.18 ± 0.14^ij^	1.39 ± 0.12^rs^	6.57 ± 0.24^w^
		*p*‐Coumaric acid	1.60 ± 0.04^a^	2.37 ± 0.20^h^	3.22 ± 0.19^p^	5.59 ± 0.01^uvw^
		7‐Hydroxycoumarin	2.50 ± 0.19^a^	3.05 ± 0.19^k^	0.87 ± 0.07^q^	3.92 ± 0.24^x^
		Ferulic acid	2.01 ± 0.14^a^	2.47 ± 0.10^ij^	3.53 ± 0.22^pq^	6.00 ± 0.30^vw^
		Rutin	20.21 ± 1.16^a^	21.02 ± 1.31^l^	0.74 ± 0.04^s^	21.76 ± 1.28^y^

*Notes.* Data are presented as means ± standard deviation (*n* = 3). “WLW” means wampee leaves wine, “FR” means free residue, “BR” means bound residue, and “TR” means total residue. Different letters in the same column of same phytochemical indicate significant differences at *p* < 0.05 from different samples. Letters group “a–g” corresponds to the row of wine, “h–n” corresponds to the row of free in residue, “o–t” corresponds to the row of bound in residue, and “u–z” corresponds to the row of total form in residue.

Vanillic acid, *p*‐coumaric acid, 7‐hydroxyl coumarin, rutin and ferulic acid of control did not be detected.

In residue, the decreasing order of free phytochemicals was: rutin > vanillic acid > 7‐hydroxycoumarin > ferulic acid > *p*‐coumaric acid while that of bound was: ferulic acid > *p*‐coumaric acid > vanillic acid > 7‐hydroxycoumarin > rutin (Table 3). Additionally, the order of total phytochemical content in residue was: rutin > vanillic acid > ferulic acid > 7‐hydroxycoumarin > *p*‐coumaric during unsealed fermentation that changed into rutin > vanillic acid > ferulic acid > *p*‐coumaric > 7‐hydroxycoumarin in sealed fermentation.

Rutin as a functional flavonoid in plants has been extensively been investigated for its antioxidant and anti‐inflammatory properties (Diwan, Brown, & Gobe, 2017). The average concentration of rutin (2.45 mg/L) was reported in 45 kinds of wine and found that it is more in red wine compared to white (Agatonovic‐Kustrin, Hettiarachchi, Morton, & Razic, 2015). In addition, an increment in rutin from 7.77 to 10.95 mg/L was reported in different maceration protocols (4–20 days) during the production of red wine (Rosenzweig et al., 2017). In the present study, significant level of rutin in WLW at 21.00 mg/100 g was detected during the first 15 days’ fermentation and then slightly decreased in the following process. Additionally, rutin content in the free fraction of residue decreased to 27.92 ± 0.62 mg/100 g on Day 6 from its initial level of 37.67 ± 2.15 mg/100 g. Afterward, slight increase up to 33.38 ± 0.88 mg/100 g was noted on Day 15. But it was decreased up to 20.17 ± 1.14 mg/100 g on Day 21 of fermentation. Conversely, rutin content in the bound fractions of residue was at low concentration about 0.80 mg/100 g during the fermentation. In the present investigation, dynamic changes in the rutin content of WLW were probably caused by the balance between leaching from leaves and native oxidation. And the decrease in rutin in the free fraction of residue might be due to the free radicals generated from mass propagation of yeasts in the unsealed fermentation, oxidation, and hydrolysis while slight increment may be related to the duration of maceration (Rosenzweig et al., 2017).

Vanillic and ferulic acids are phenolic acids, while *p*‐coumaric acid belongs to phenylpropanoic acids and 7‐hydroxycoumarin is one of the polyphenolic components (Li et al., 2017; Yrbas, Morucci, Alonso, & Gorzalczany, 2015). It has been reported that in wine, these phytochemicals are involved in the interaction between flavor substances and oral sensation (Esteban‐Fernández, Muñoz‐González, Jiménez‐Girón, Pérez‐Jiménez, & Pozo‐Bayón, 2018). In a recent study, considerable amount of vanillic, ferulic, and *p*‐coumaric acids has been reported in Montenegrin *Vranac* red wine (Đorđević et al., 2018). In WLW, substantial enhancement of vanillic acid content was observed during the process of whole fermentation. Likewise, an increasing trend was also noted in ferulic acid and *p*‐coumaric acid concentration during wine‐fermenting, whereas 7‐hydroxycoumarin was almost stable (2.45 mg/100 g).

In the free fraction of residue, vanillic and ferulic acids were stable, while an increasing trend was noted for *p*‐coumaric acid. However, 7‐hydroxycoumarin decreased to 3.77 ± 0.09 mg/100 g on Day 3 from its initial level of 4.25 ± 0.34 mg/100 g in unsealed fermentation. A gradual increase in the concentration of 7‐hydroxycoumarin up to 4.56 ± 0.13 mg/100 g was noted on Day 15 but it decreases again to 3.05 ± 0.19 mg/100 g on Day 24. In the case of bound fraction of residue, vanillic acid and 7‐hydroxycoumarin showed roughly stable and low concentrations during the whole time. Conversely, ferulic acid and *p*‐coumaric acid depicted increasing tendency during the fermentation process. Fluctuation in the levels of these compounds probably related to substantially microbial activities, mutative environment with hydro‐alcoholic medium and degradation.

### 3.4 Dynamic changes in the antioxidant activity of WLW and residue

Antioxidant potential of the WLW and residue was determined by PSC assay, which is a popular method for assessing total antioxidant activity in food extracts due to its rapidity and high sensitivity (Guo et al., 2017; Wang, Sun, et al., 2015; Wang, Wang, et al., 2015). The variations in the antioxidant activity of WLW and residue estimated by PSC assay were presented in Table 4. For WLW, the PSC value ranged from 29.98 ± 1.69 to 67.21 ± 2.41 μmol ASA equiv./100 g on Day 0 and Day 12, respectively. These values were significantly different at *p *<* *0.05. Our results indicated that the fermentation process enhances antioxidant potential of fermented foods, particularly maintained for 12 days would be the best for making health beneficial WLW. For residue, the highest PSC value of free fraction was 123.6 ± 7.5 μmol ASA equiv./100 g and for bound was 21.62 ± 1.78 μmol ASA equiv./100 g on Day 15 and Day 9, respectively. However, PSC value was lowest on Day 9 and Day 24 for free (66.47 ± 4.83 μmol ASA equiv./100 g) and bound fractions (17.08 ± 0.81 μmol ASA equiv./100 g). Likewise, total PSC value in residue was at the peak on Day 0 (142.5 ± 6.3 μmol ASA equiv./100 g), while lowest was calculated on Day 9 (88.10 ± 3.83 μmol ASA equiv./100 g).

**Table 4:** Antioxidant activities (μmol ASA equiv./100 g) of WLW and residue

Sample	Unsealed fermentation		Sealed fermentation						
	Day 0	Day 3	Day 6	Day 9	Day 12	Day 15	Day 18	Day 21	Day 24
WLW	29.98 ± 1.69^f^	46.55 ± 14.52^e^	49.61 ± 1.27^de^	54.74 ± 4.44^bcde^	67.21 ± 2.41^a^	61.99 ± 3.71^ab^	60.09 ± 2.03^abc^	57.25 ± 1.41^abcd^	50.79 ± 1.72^cde^
FR	123.4 ± 6.4^h^	101.9 ± 5.9^ij^	100.8 ± 6.9^ij^	66.47 ± 4.83^k^	75.54 ± 7.62^k^	123.6 ± 7.5^h^	112.2 ± 6.3^i^	108.9 ± 2.6^i^	93.61 ± 6.41^j^
BR	M, 19.10 ± 0.58^pq^	19.12 ± 1.63^pq^	19.78 ± 1.21^op^	21.62 ± 1.78^o^	18.65 ± 1.58^pq^	18.68 ± 1.60^pq^	17.94 ± 1.54^pq^	18.11 ± 0.88^pq^	17.08 ± 0.81^q^
TR	142.5 ± 6.3^u^	121.0 ± 7.2^vw^	120.6 ± 5.8^vw^	88.10 ± 3.83^x^	94.18 ± 8.97^x^	142.3 ± 8.0^u^	130.1 ± 7.4^v^	127.0 ± 3.4^v^	110.7 ± 6.0^w^

*Notes*. Data are presented as means ± standard deviation (*n *= 3). “WLW” means wampee leaves wine, “FR” means free residue, “BR” means bound residue, and “TR” means total residue. Different letters in the same row indicate significant differences at *p* < 0.05 from different samples. Letters group “a–g” corresponds to the row of wine, “h–n” corresponds to the row of free in residue, “o–t” corresponds to the row of bound in residue, and “u–z” corresponds to the row of total form in residue. PSC value of control did not be detected.

A “decrease‐increase‐stable” fluctuation in antioxidant activity has been reported during pomegranate wine‐fermenting, which could be associated with variations in polyphenols (Lan et al., 2017). While a rapid increase in the early 2 days and unobvious alteration in the antioxidant activity was observed in next 8 days in mulberry wine‐making (Wang, Sun, et al., 2015; Wang, Wang, et al., 2015). An “increase‐roughly stable‐slightly decrease” changes were observed in the total antioxidant activity of WLW in this study.

The PSC value and phytochemical of WLW depicted better association determined by Pearson's correlation analysis such as: vanillic acid: *R* ^2^ = 0.71, *P *<* *0.05; *p*‐coumaric acid: *R* ^2^ = 0.68, *P *<* *0.05; 7‐hydroxycoumarin: *R* ^2^ = 0.76, *P *<* *0.05; ferulic acid: *R* ^2^ = 0.74, *P *<* *0.05). However, rutin showed week relation with PSC (*R* ^2^ = 0.119, *P *=* *0.87). Though, results of Iacopini (Iacopini, Baldi, Storchi, & Sebastiani, 2008) showed strong association between antioxidant activity and rutin but in the present study rutin expressed an awful correlation with PSC value regardless of its dominant concentration. In our work, the variation in the total antioxidant activity of WLW was consistent with mulberry wine (Wang, Sun, et al., 2015; Wang, Wang, et al., 2015), and almost 2‐fold increase of total antioxidant activity was observed from Day 0 to Day 21 during the wine‐making. Besides, PSC value was maintained stable at 60.00 μmol ASA equiv./100 g up to 9 days after 12‐day alcoholic fermentation. Therefore, 12 days’ fermentation could be a useful point for making health beneficial wine from wampee leaves.

## 4 CONCLUSION

Present study was aimed to excavate the application of wampee leaves product in food industries. Our study revealed that wampee leaves wine (WLW) could be a better choice to promote consumer health. Because fermentation process has significantly enhanced the concentration of alcoholic compounds, polyphenolics, and antioxidant capacity of WLW. Additionally, highest levels of phenolic and flavonoid content and antioxidant capacity in WLW were observed during fermentation, particularly on Day 12. Therefore, we suggest that 12 days’ fermentation could be useful for making health beneficial wine from wampee leaves.

## CONFLICT OF INTERESTS

The authors declare no conflict of interest.
